# Twisting, untwisting, and retwisting of elastic Co-based nanohelices

**DOI:** 10.1038/s41467-023-40001-w

**Published:** 2023-07-22

**Authors:** Wei Du, Feng Gao, Peng Cui, Zhiwu Yu, Wei Tong, Jihao Wang, Zhuang Ren, Chuang Song, Jiaying Xu, Haifeng Ma, Liyun Dang, Di Zhang, Qingyou Lu, Jun Jiang, Junfeng Wang, Li Pi, Zhigao Sheng, Qingyi Lu

**Affiliations:** 1 State Key Laboratory of Coordination Chemistry, School of Chemistry and Chemical Engineering, Collaborative Innovation Center of Advanced Microstructures, Nanjing National Laboratory of Microstructures, Nanjing University, 210023 Nanjing, P. R. China; 2 State Key Laboratory of Materials-Oriented Chemical Engineering, College of Chemical Engineering, Nanjing Tech University, 211816 Nanjing, P. R. China; 3 Department of Materials Science and Engineering, Jiangsu Key Laboratory of Artificial Functional Materials, Collaborative Innovation Center of Advanced Microstructures, College of Engineering and Applied Science, Nanjing University, 210023 Nanjing, P. R. China; 4 Hefei National Laboratory for Physical Sciences at Microscale and Anhui Laboratory of Advanced Photon Science and Technology, University of Science and Technology of China, 230026 Hefei, AnHui P. R. China; 5 High Magnetic Field Laboratory, CAS Key Laboratory of High Magnetic Field and Ion Beam Physical Biology, Hefei Institutes of Physical Science, Chinese Academy of Sciences, 230031 Hefei, Anhui P. R. China; 6 Anhui Province Key Laboratory of Condensed Matter Physics at Extreme Conditions, High Magnetic Field Laboratory and High Magnetic Field Laboratory of Anhui Province, HFIPS, Chinese Academy of Sciences, 230031 Hefei, Anhui P. R. China

**Keywords:** Synthesis and processing, Structural properties, Organic molecules in materials science

## Abstract

The reversible transformation of a nanohelix is one of the most exquisite and important phenomena in nature. However, nanomaterials usually fail to twist into helical crystals. Considering the irreversibility of the previously studied twisting forces, the reverse process (untwisting) is more difficult to achieve, let alone the retwisting of the untwisted crystalline nanohelices. Herein, we report a new reciprocal effect between molecular geometry and crystal structure which triggers a twisting-untwisting-retwisting cycle for tri-cobalt salicylate hydroxide hexahydrate. The twisting force stems from competition between the condensation reaction and stacking process, different from the previously reported twisting mechanisms. The resulting distinct nanohelices give rise to unusual structure elasticity, as reflected in the reversible change of crystal lattice parameters and the mutual transformation between the nanowires and nanohelices. This study proposes a fresh concept for designing reversible processes and brings a new perspective in crystallography.

## Abstract (web summary)

The reversible transformation of a nanohelix is one of the most exquisite and important phenomenon in nature but nanomaterials usually fail to twist into helical crystals or untwist. Here, the authors report a reciprocal effect between molecular geometry and crystal structure which triggers a twisting-untwisting-retwisting cycle for tri-cobalt salicylate hydroxide hexahydrate.

## Introduction

Nature has unique forces to create exquisite architectures from nanoscale to macroscale. The helical conformation is one of the most representative examples, which broadly exists in biological systems, spanning from the collagen triple helix to the macroscopic seashell^1–4^. The double helix structure of a DNA chain is known to play crucial roles in the storage and reproduction of genetic information and is believed to be the foundation of life and modern biology^5–7^. Stimulated by the complexity and significance of natural helices, scientists have made great efforts to mimic this important configuration, and created novel and functional helical structures^8–13^.

The direct link between superstructure and molecular structure has been reported in many cases. However, the attempt to establish correlations between the macroscopic chiral morphology and the molecular configuration has not been very successful so far^14^. The reported twisting processes are usually triggered by defects, mechanical forces, or physical/chemical fields^6,12–19^. Spherulites with twisted mesoscale fibers can grow from slightly undercooled melts^13–16^, and soft materials (including elastomers and hydrogels) with heterogeneous structures^20–23^ undergoing differential swelling/shrinkage makes bending or folding deformations occur^24–27^. Some twisting processes were thought to be accompanied by conformational changes of molecules^28^. The surface stress coming from the difference of the bonding patterns and molecular conformations on surface, and the intrinsic stresses in crystal structures possibly liberated at small sizes might be the key points for the intrinsically twisted morphology^28^. The geometric frustration played an important role in the formation of twisted molecular crystals whose macroscopic pitch lengths continuously varied with crystal size^29^.

Nanohelices are crucial for constructing future nanodevices, as confirmed by various macroscopic helix components such as gears, springs, and propellers^30–38^. Their fabrication has been believed to be induced by effects such as diverged electrostatic energy of polar surfaces along wide nanoribbons, the selective adsorption of racemic polymers on crystal surfaces, the hierarchical self-assembly of dipeptides, and the geometric incompatibility of different types of chains^39–50^. Although the helical nanostructure is a representative and important geometry and has become a research frontier in materials science, research reports on helical nanocrystals are still limited. Also, the capture of the twisting process and the control of helix growth are quite difficult to realize. How the molecules arrange and interact to form helix geometry remains a mystery.

The research on untwisting relies on the formation of helices, which makes the research on untwisting more rare^51^. The uncoiling of nanohelices is seldom reported except famous untwisting processes created by nature. For soft heterogeneous matter, such as a bi-layer gel in which a responsive gel sheet is bound to a nonresponsive one, the curvatures change under different stimulations^21,22,27^. Recently, the untwisting processes of a few helices, including hippuric acid^12,14,52,53^ and δ-mannitol^16^, have been reported. Snapshots of optical movies about the growth of hippuric acid needle clearly showed that the twisting dynamic depended on the thickness. The twisting at sharp tip was more effective, and the crystal unwound as it got thicker^52,53^. So far, how to reverse the initial twisting process and realize the untwisting of crystalline nanohelices has remained a big challenge as the previously studied twisting forces are usually irreversible. Not to mention the retwisting of the untwisted crystalline nanohelices; success would mean repeatable mutual conversion between the nanohelices and the straight nanowires. Therefore, such a reversible transformation has long been considered infeasible, and no related work has been reported.

Herein, a twisting–untwisting–retwisting cycle in a Co-based complex, tri-cobalt salicylate hydroxide hexahydrate, has been firstly realized, triggered by a mutual effect between the molecular geometry and the crystal structure. Theoretical calculations and various characterizations demonstrate that the twisting impetus comes from competition between the condensation reaction and stacking process, which differs from the previously reported twisting mechanisms. Moreover, a special kind of “elastic” nanohelical structure has also been presented, which has reversible lattice parameters and can mutually transform between the nanowires and nanohelices. These nanohelices show improved properties in magnetism and electrocatalysis, and the twisting process can be extended to Ni- and Ni/Co-based complexes and the corresponding polycrystalline inorganic oxide nanohelices through further conversions. This work provides a new perspective for crystallography research and opens a door for designing various “elastic” structures and reversible transformations relying on the precise adjustment of molecular interaction and crystal configuration.

## Results

**Fig. 1:**
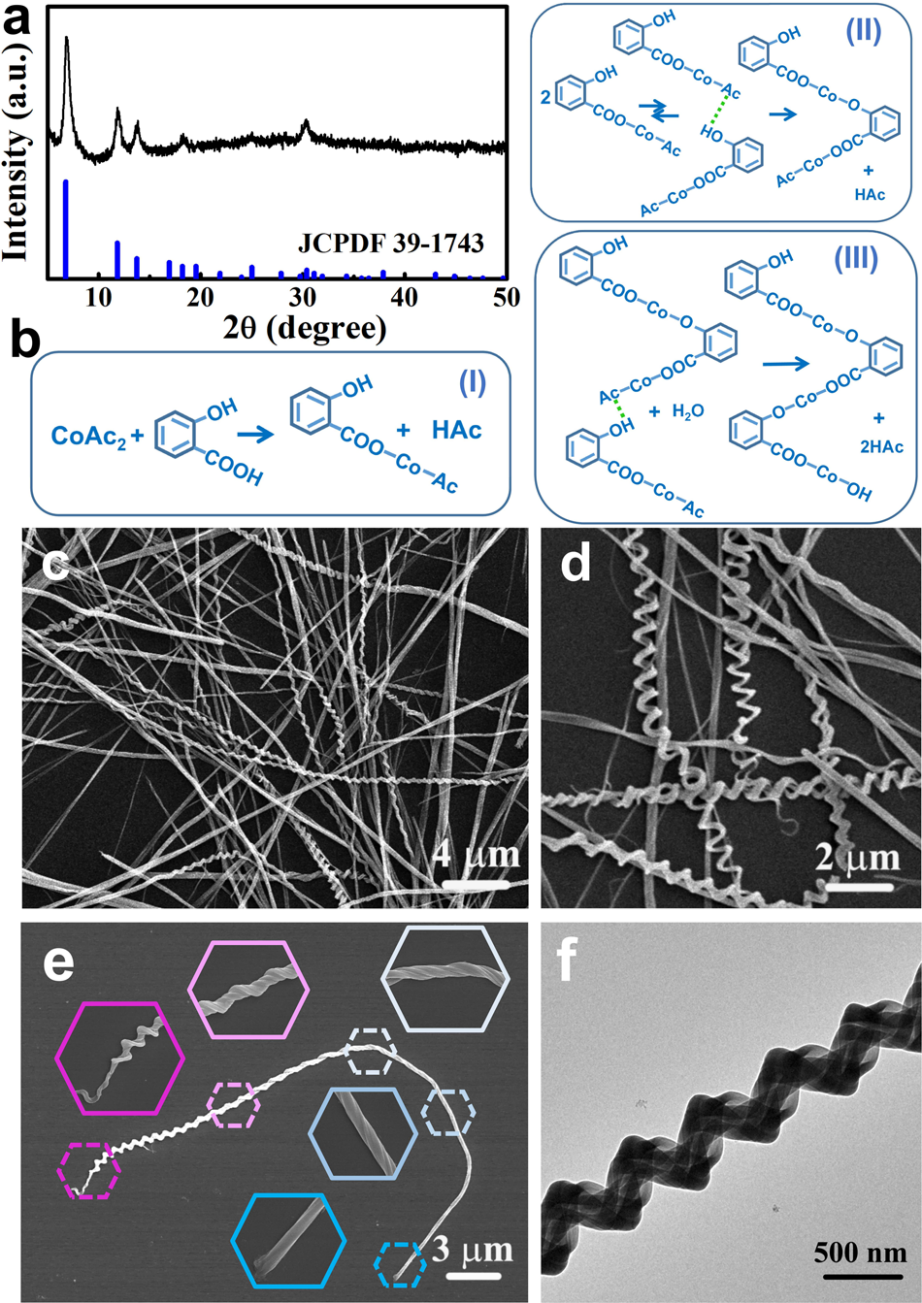
Nanohelices synthesized under ethanol-thermal conditions at 80 °C for 4 h. **a** XRD pattern; **b** reaction equations; **c**, **d** SEM images with low and high magnifications; **e** SEM image of a nanohelix (insets: magnifications of different segments); **f** TEM image.

Ortho-hydroxybenzoic acid, known as salicylic acid, has two different functional groups, both of which can coordinate with metal ions. With salicylic acid as the coordinating agent, we successfully synthesized tri-cobalt salicylate hydroxide hexahydrate nanohelices under 80 °C ethanol-thermal conditions for 4 h (See “Methods” for experimental details). This simple reaction occurs under mild conditions, requiring neither surfactants, templates, or substrates, nor high-temperature processes. Figure 1a shows X-ray diffraction (XRD) pattern of the as-synthesized nanohelices, confirming the formation of tri-cobalt salicylate hydroxide hexahydrate^54^. The reaction equation is presented in Fig. 1b. Scanning electron microscopy (SEM) images (Fig. 1c, d) indicate that the product consists of twisting nanostructures with diameters of a few hundred nanometers and lengths up to ~40 μm. The numbers of left-twisting and right-twisting structures do not differ much. This kind of nanohelix is not twisted in the same way from one end to the other, which can be observed more clearly in Fig. 1e (the insets show the enlarged segment details of the nanohelix). More SEM images are presented in Supplementary Fig. 1. Some helices are composed of a single twisted nanowire (Supplementary Fig. 1a), while some incorporate several twisted nanowires (Supplementary Fig. 1g). Transmission electron microscopy (TEM) image of a helical structure is shown in Fig. 1f.

**Fig. 2:**
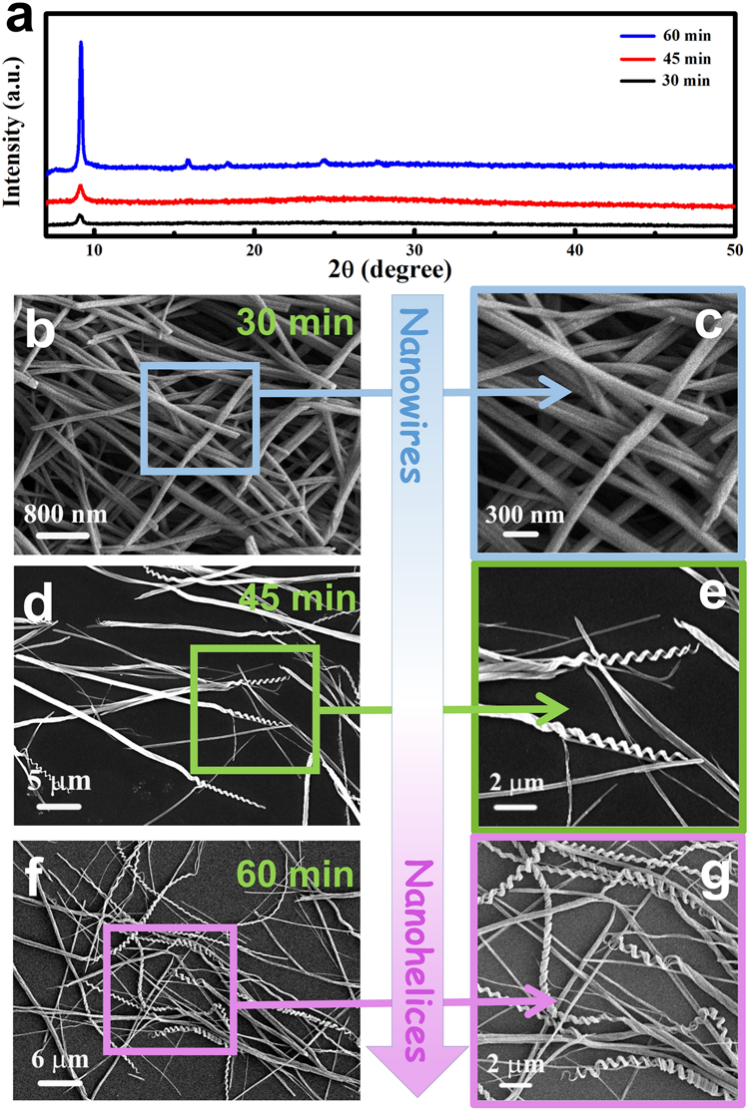
XRD patterns and SEM images of the products synthesized under 80 °C ethanol-thermal conditions for different times. **a** XRD patterns, **b**, **c** 30 min, **d**, **e** 45 min, and **f**, **g** 60 min.

During the growth process, the reactant, solvent, reaction temperature, and time were undoubtedly important. When salicylic acid was replaced by pyrocatechol, m-hydroxybenzoic acid, phthalandione or salicylaldehyde, the products were found to be nonhelical particles (Supplementary Fig. 2), or no precipitates formed at all. When Co salt and salicylic acid were mixed in alkaline (KOH) aqueous solution, tri-cobalt salicylate hydroxide hexahydrate precipitated quickly at room temperature with a rod-like morphology. The experimental details, XRD pattern and SEM images of the formed nanorods are shown in Supplementary Method 1 and Supplementary Fig. 3. When Co salt and salicylic acid were mixed in ethanol, the reaction rate sharply slowed down. To produce the complex precipitate, Co salt must react with salicylic acid under 80 °C ethanol-thermal conditions for more than 20 min. Figure 2 shows the XRD patterns and SEM images of the products prepared under 80 °C ethanol-thermal conditions for 30, 45, and 60 min. The previous literature reported that the product’s crystallinity usually increases as the reaction proceeds^55,56^, and our XRD characterizations also confirm that from 30 to 45 to 60 min, the crystallinity of the product increases (Fig. 2a). However, the morphology change is surprising. The product for the 30-min reaction has wire-like morphology (Fig. 2b, c). As the reaction went on, the nanowires twisted gradually (Fig. 2d, e). For the 60-min reaction, the product consists of nanohelices (Fig. 2f, g). These results give a qualitative suggestion that the nanohelices are transformed from nanowires as the reaction proceeds.

**Fig. 3:**
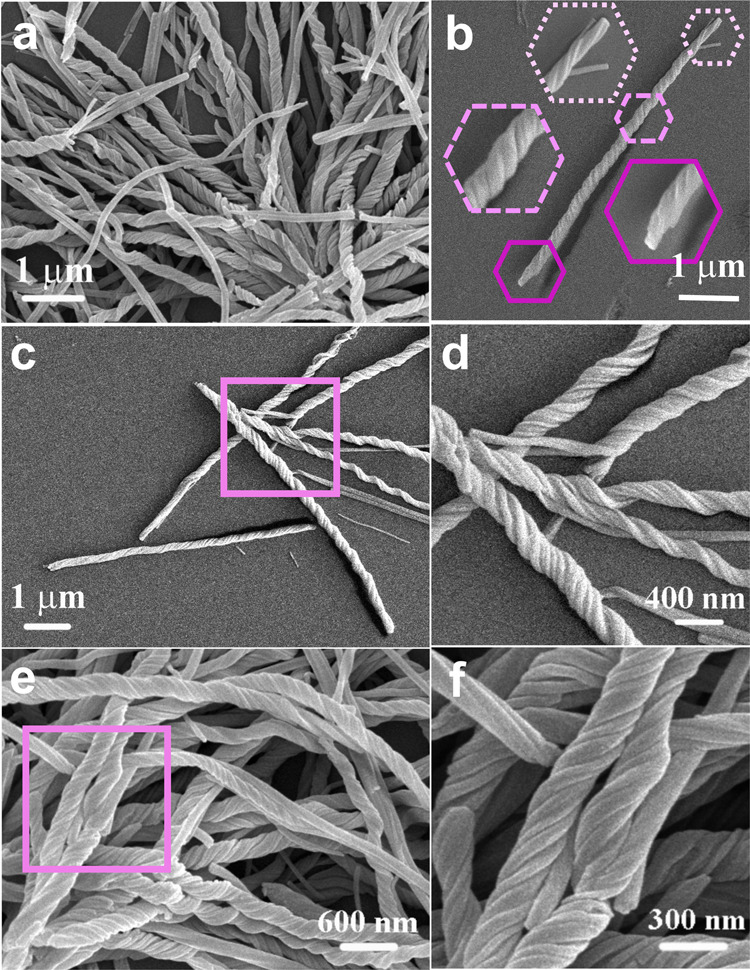
Nanohelices prepared with the addition of hexadecylamine. **a** SEM image with a low magnification; **b** SEM image of a nanohelix (insets: magnifications of the different segments); **c**–**f** SEM images and their high magnifications for the selected areas.

The gradual transformation from nanowires to nanohelices brings up the possibility of intervening in the twisting process. We then regulated the growth process by adding a surfactant, such as polyvinyl pyrrolidone (PVP) or triblock copolymer P123, into the reaction system. Interestingly, with the addition of hexadecylamine, the produced nanohelices, as shown in Fig. 3, are shorter, and usually twisted from one end all the way to the other end, which is seen more clearly in Fig. 3b (the insets show the enlarged segment details of the nanohelix). The experimental details and more SEM images are shown in Supplementary Method 2 and Supplementary Fig. 4.

**Fig. 4:**
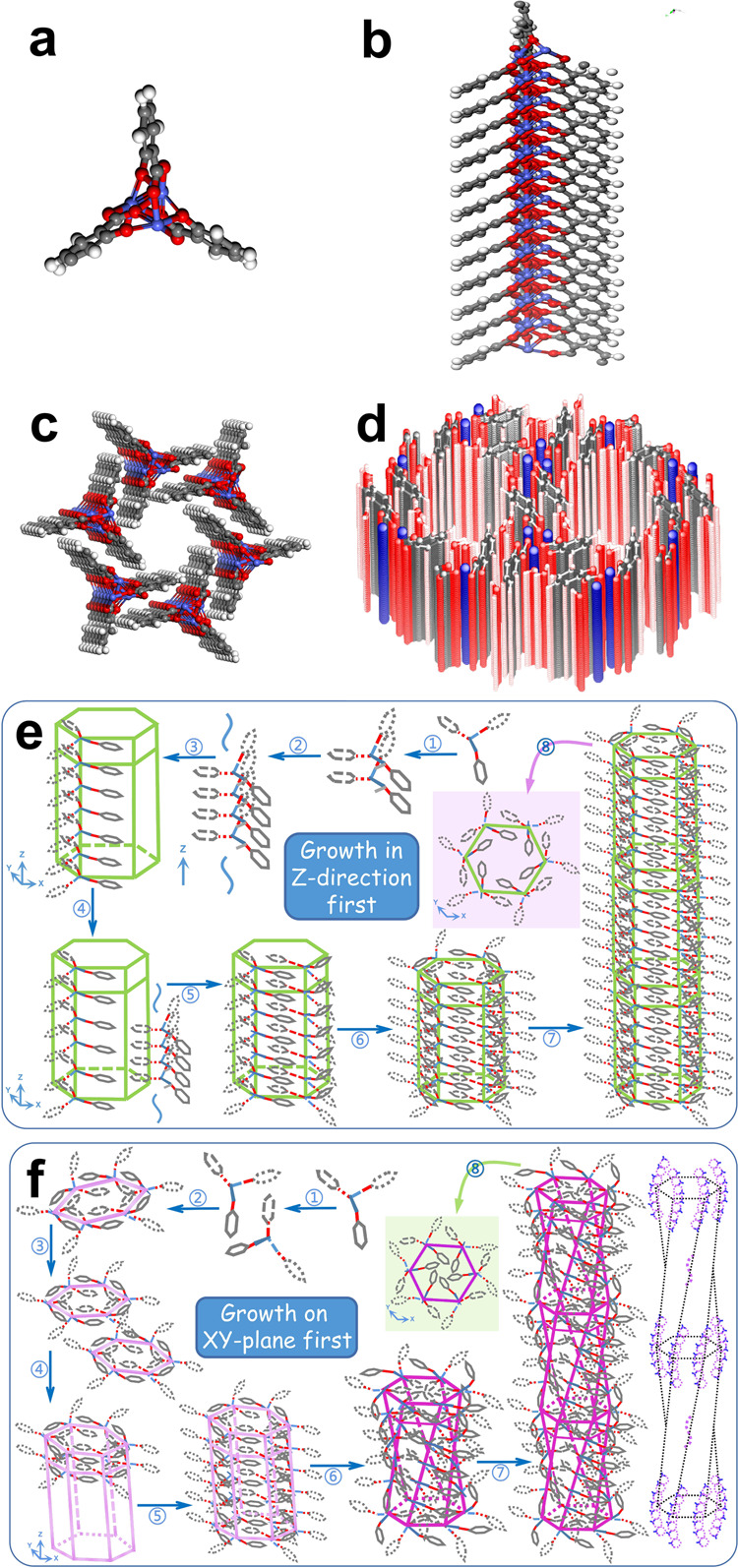
Model for theoretical calculations and growth mechanisms. **a** Triangular monomer; **b** triangular monomers connect head-to-tail to form a long chain; **c** six long chains assemble to form a hexagonal prism structure; **d** crystal structure model; **e** nanorod growth mechanism; **f** nanohelix growth mechanism.

To investigate the twisting mechanism, we first used density functional theory (DFT) to establish a molecular model. The computational methods are described in Supplementary Method 6. Theoretical calculations suggest that a tri-cobalt salicylate hydroxide hexahydrate monomer takes on a triangular structure (Fig. 4a), in which the optimized Co–Co bonding length (3.06 Å) agrees well with the measured extended X-ray absorption fine structure (EXAFS) result (3.04 Å) (See detailed information in Supplementary Method 7, Supplementary Fig. 9, and Supplementary Table 1). Many such triangle monomers connect in a head-to-tail style through the condensation process, forming a chain along the growth direction (Fig. 4b). Six such long chains assemble and gradually form a hexagonal prism (Fig. 4c). The intermolecular distance, calibrated as the distance between the symmetry centers of the two almost in-parallel benzene rings, is about 3.4 Å, indicating the existence of offset face-to-face (F-type) π–π interaction^57,58^. The final structure has the rod-like configuration shown in Fig. 4d. Using this model, the infrared (IR) spectrum and XRD pattern were calculated, and the calculated results agree well with the corresponding measured results (Supplementary Fig. 5).

Based on the above model, the formation of nanorods is predictable. In a KOH aqueous solution, the condensation process proceeds very quickly due to the accelerating effect of the alkaline solution^59^. Long straight chains form at first and then assemble into hexagonal prisms through the F-type π–π interaction between the laterally adjacent benzene rings, as the theoretical model shows in Fig. 4a–d. The final product has a rod shape (Supplementary Fig. 3). In this case, subject to the rigidity of the straight chains, the adjacent benzene rings cannot pack more tightly to reach stronger π–π interaction. A schematic of the nanorod growth is shown in Fig. 4e.

The growth mode in ethanol is different. The lack of alkaline solution and the use of ethanol as solvent obviously slow down the condensation process, which prohibits the quick formation of the monomer chains at the very beginning. The complex molecules gradually assemble into a hexagonal planar structure first through π–π stacking. Without the limitation of a straight chain, benzene rings of two adjacent complex molecules tend to twist a little to assemble more tightly to realize stronger π–π interaction, edge-to-face (T-type) π–π stacking^60^, which leads to a hexagonal planar structure somewhat smaller and distorted. Such hexagonal planar structures then arrange themselves face-to-face to condense with each other. Subject to the rigidity of the tightly assembled but slightly distorted hexagonal planar structure, a mismatch arises between the hexagonal plane and the plane above or below it, gradually leading to the formation of a nanohelix. Such twisting is mainly due to the competition between the condensation process for the monomer chain and the stacking process for the hexagonal planar structure. We call this a competition twisting mechanism, showing its schematic in Fig. 4f.

According to the above competition twisting mechanism, the nanohelix and nanorod share the same triangular building blocks, having same chemical valence and cobalt coordination number. However, the interaction and arrangement patterns between triangular structures in the nanohelix are different from those in the nanorod. For example, the former should have stronger π–π interactions, smaller lattice spacings and a more complicated Co-structural environment. To examine the twisting mechanism, various characterizations were employed to investigate the as-synthesized nanohelices and nanorods. Their IR and Raman spectra (Supplementary Figs. 6 and 7) look almost the same, and their X-ray photoelectron spectroscopy (XPS) (Supplementary Fig. 8) and EXAFS (Supplementary Fig. 9) results also look alike, which confirms the presence of the same triangular building blocks in nanorods and nanohelices. The differences in nuclear magnetic resonance (NMR), electron paramagnetic resonance (EPR) and terahertz (THz) spectra, on the other hand, reflect the dissimilar intermolecular interactions and arrangements between the two structures.

**Fig. 5:**
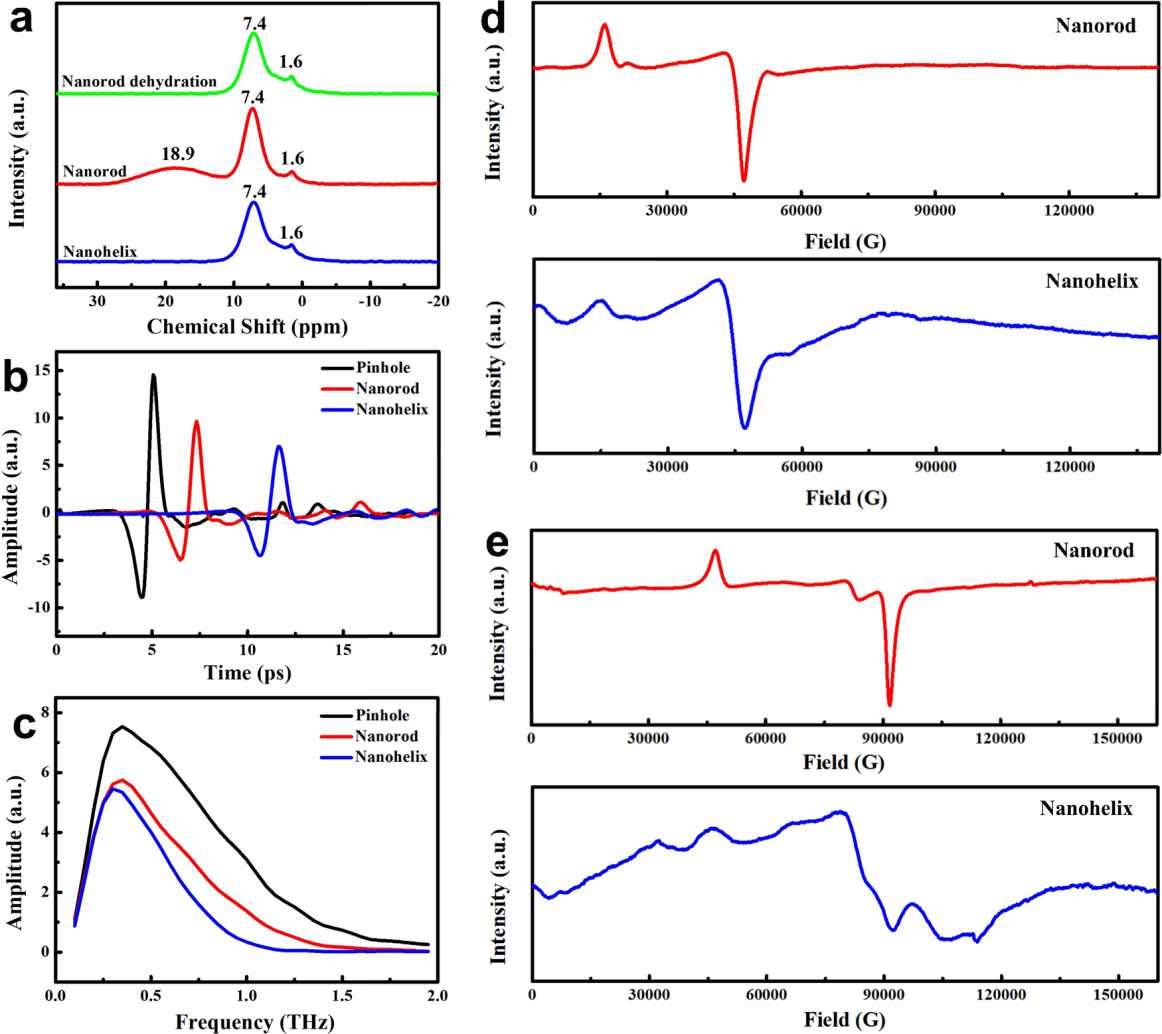
NMR, THz, and EPR spectra. **a** Solid-state ^1^H NMR spectra of the nanorods, nanohelices and nanorods after dehydration at 180 °C; **b** THz time-domain spectra and **c** THz frequency-domain spectra of the nanorods and nanohelices (pinhole as the background/reference); **d**, **e** EPR spectra of the nanorods and nanohelices excited at a frequency of 212 and 428 GHz.

Weak intermolecular interactions (such as hydrogen bonds and π–π interactions) between triangular building blocks may be essential in the formation of nanohelix and nanorod structures^43,54^. The solid-state ^1^H NMR spectra (Fig. 5a) reveal that water-mediated hydrogen bonding exists in the nanorods, which can be removed by heating the sample at 180 °C for 3 h. Such water hydrogen bonding cannot be found in the nanohelices, suggesting that water hydrogen bonding is not essential during the twisting process.

THz spectroscopy is a useful technique to investigate weak molecular interactions relying on their characteristic absorptions in the terahertz band^61,62^. Figure 5b, c shows the THz time- and frequency-domain spectra of the nanorods and nanohelices. It can be observed that for both the nanorods and nanohelices, the terahertz signal has a time delay and becomes significantly attenuated, indicating that weak interactions exist in both. However, the nanohelices have more significant time delay and stronger absorption, revealing that the weak molecular interactions in the nanohelices are stronger than those in the nanorods^63,64^. This result, combined with the NMR data, indicates that the π–π interactions are stronger in the nanohelices than in the nanorods, which supports the competition twisting mechanism.

EPR spectroscopy was also utilized to investigate the difference in the Co(II) coordination environment between the nanorods and nanohelices. Figure 5d shows the EPR spectra of the two excited at a frequency of 212 GHz. Their resonance peak positions are almost identical, indicating similar spin-level splitting of Co(II) in both samples^65^. However, the resonance peaks of the nanohelices are obviously wider, showing that the spin interactions between Co(II) in the nanohelices are more complicated than those in the nanorods^65^, which is consistent with the twisting cobalt chain in the nanohelix.

In general, the higher the excitation frequency, the better the resolution of the g-value anisotropy. So to further explore the differences between the nanorods and nanohelices, we measured their EPR spectra with a higher excitation frequency (428 GHz, Fig. 5e). Besides the dissimilarity in the peak width, another obvious difference is observed. The nanohelices have two resonance peaks which are missing for the nanorods. These suggest a decrease in the symmetry of the Co(II) coordination environment in the nanohelices and a slight change in the Co(II) coordination environment after twisting^66^. Based on the competition twisting mechanism, in a nanohelix, the intermolecular Co(II) environment is slightly different because of the twisting between two hexagonal planar structures during the condensation process. This change decreases the symmetry of the coordination structure and brings up new resonance peaks. Therefore, this EPR study also provides supporting evidence for the competition twisting mechanism.

**Fig. 6:**
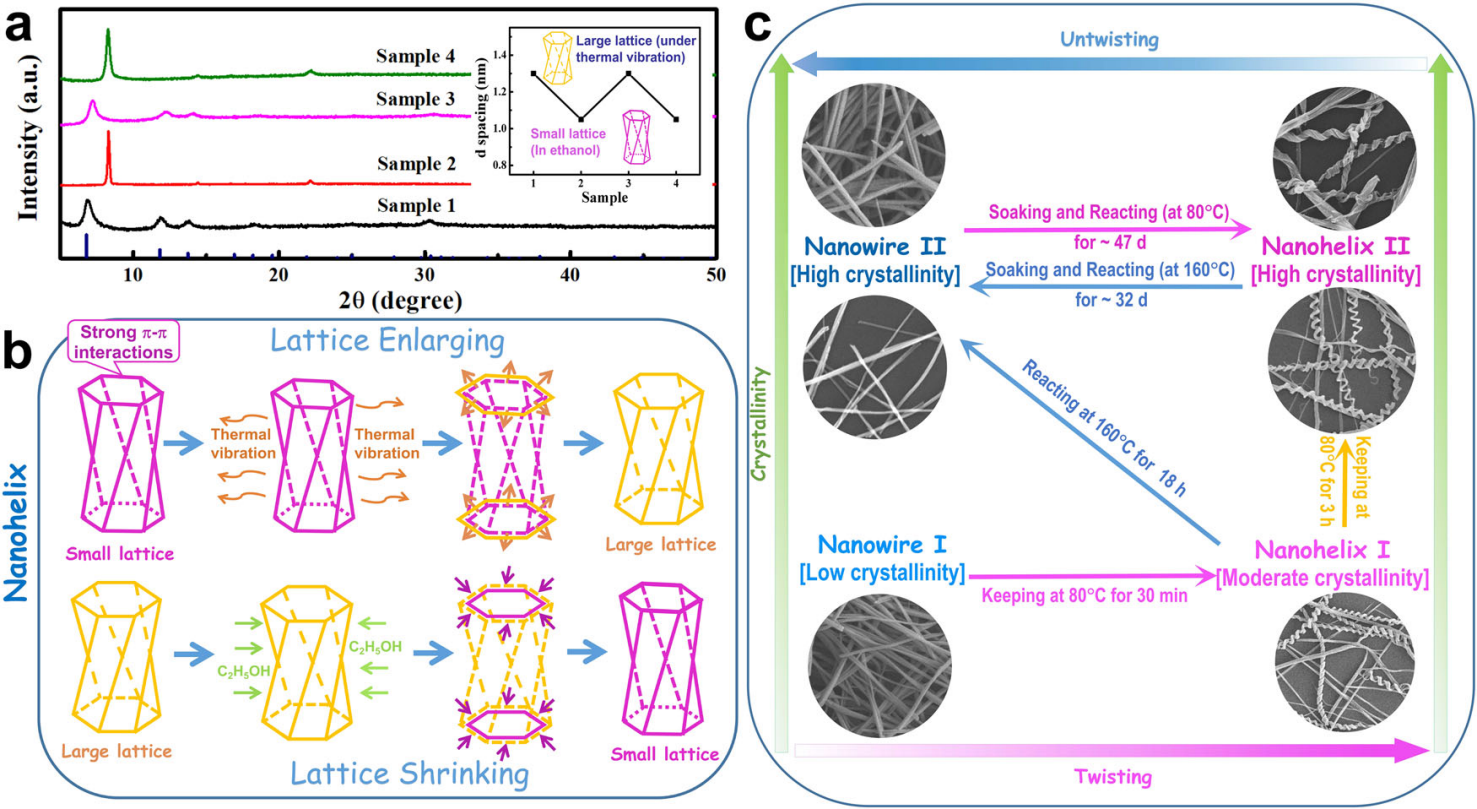
Reversible change of nanohelices. **a** XRD patterns of the samples kept under different external conditions. Inset: changes in the d-spacing of the strongest diffraction peak. Sample 1: as-synthesized nanohelices; Sample 2: Sample 1 kept in ethanol at room temperature for 24 h and then dried naturally; Sample 3: Sample 2 kept in ethanol solution and then dried in 80 °C oven for 3 h; Sample 4: Sample 3 kept in ethanol at room temperature for 24 h and then dried naturally; **b** crystal lattice transformation schematic of a nanohelix with the stated external conditions; **c** mutual transformation processes between the nanowires and nanohelices.

The produced nanohelices were found to have a special “elastic” crystal lattice, probably due to the special twisting mechanism. The XRD characterizations (Fig. 6a) reveal that as the external conditions change, the crystal lattice parameters of the nanohelices can vary back and forth. When the nanohelices are kept in ethanol for 24 h and dried naturally, their interplanar spacings shrink; for example, the d spacing of the strongest diffraction peak shrinks from 13.0 to 10.5 Å. This variation is reversible (Fig. 6a inset): after this sample is kept in ethanol solution and then dried at 80 °C for 3 h, the d spacing expands back to 13.0 Å. This phenomenon does not exist in the nanorods. Instead, their crystal structure collapses after they are kept in ethanol for 24 h, as confirmed by the XRD patterns shown in Supplementary Fig. 10. This kind of “elastic” crystal lattice must come from the material’s microstructure and is related to the intermolecular interaction. A schematic of the lattice change for the nanohelices is shown in Fig. 6b. The lattice “elastic” change encourages us to speculate that the nanohelices synthesized dried naturally in air might have smaller interplanar spacings. The XRD characterization reveals that they do have the smaller interplanar spacings: 10.5 Å for the strongest diffraction peak (Supplementary Fig. 11). This result supports the tightly packed hexagonal-planar-structure model for the nanohelices, providing direct evidence for the competition twisting mechanism.

The above theoretical calculations, characterizations, and experimental results not only demonstrate a competition twisting mechanism (stemming from the molecular interaction and crystal geometry, unlike previously reported twisting mechanisms), but also exhibit a special kind of helical structure with a remarkable characteristic of “elasticity” (as reflected in the reversible transformations which have not been reported before). The reversible d-spacing change mentioned above is one example. Another example is the mutual transformation between the nanowires and nanohelices.

In the field of biology, twisting and the reverse untwisting process are outstanding works of nature and usually appear in pairs. However, in nanomaterials science, the twisting process is quite difficult to realize, and the untwisting process is even more rare. Not to mention the retwisting process of the straightened nanowires, which requires the free switching of the twisting force. In our experiments, under 80 °C ethanol-thermal conditions with the reaction time elongated, the product changes from nanowires with low crystallinity (30-min product denoted as *Nanowire I*, Fig. 2b, c) to nanohelices with moderate crystallinity (60-min product denoted as *Nanohelix I*, Fig. 2f, g) and then to nanohelices with high crystallinity (4-h product denoted as *Nanohelix II*, Fig. 1). It is a gradually twisting process starting from the nanowires and stemming from the competition twisting mechanism, which makes the extremely difficult untwisting process possible.

Previous references reported that the system temperature could not only change the piling pattern of the π–π interaction but also weaken or even break the π–π interaction^67,68^. Inspired by this suggestion, we designed a thermal-control procedure by putting the nanohelices under ethanol-thermal conditions but at a higher reaction temperature for a longer reaction time to adjust the π–π interaction and untwist the nanohelices. However, the experimental results reveal that under 160 °C ethanol-thermal conditions, even for 18 h, the transformation from *Nanohelix II* back to nanowires is not very successful, probably because the crystallinity of *Nanohelix II* is high, and so is its rigidity. Compared with *Nanohelix II*, *Nanohelix I* (produced with a shorter reaction time) also has the helical structure but with lower crystallinity. The experimental results (Supplementary Method 3 and Supplementary Fig. 12) reveal that the transformation from *Nanohelix I* to nanowires is successful. The sample synthesized under 160 °C ethanol-thermal conditions for 18 h with *Nanohelix I* being the reactant consists of straightened nanowires with diameters of a few hundred nanometers and lengths up to tens of micrometers (denoted as *Nanowire II*).

The fact that the transformation from *Nanohelix I* to nanowires is easier than that from *Nanohelix II* indicates that high crystallinity hinders crystal transformation. The straightened nanowires (*Nanowire II*) also have high crystallinity due to the high reaction temperature and long reaction time, making the retwisting process that follows more difficult. This problem could be resolved by greatly extending the reaction time (Supplementary Method 4). SEM images (Supplementary Fig. 13) reveal that the sample obtained by soaking *Nanowire II* in ethanol for 40 days and then treating it under 80 °C ethanol-thermal conditions for 7 days shows the helical structure again. The retwisting process is a slow and gradual process and the left- and right-twisting structures also appeared simultaneously in the product. This result demonstrates the realization of the retwisting process from nanowires with high crystallinity, where those nanowires are straightened nanohelices.

This strategy can be further confirmed by the successful straightening of *Nanohelix II*. As mentioned above, we failed to realize the untwisting of *Nanohelix II* in our first attempt. By greatly extending the reaction time, we found that the nanohelices with high crystallinity (*Nanohelix II*) can also be transformed into the nanowires successfully, as confirmed by the product’s SEM images (Supplementary Fig. 14). Thus, transformation between nanowires and nanohelices has been realized. The whole nanowire-nanohelix transformation schematic is shown in Fig. 6c. The transformation between the nanowires and nanohelices occurs at a suitable temperature (low temperature for the nanohelices and high temperature for the nanowires), and the reaction time should be very long when the crystallinity is high.

**Fig. 7:**
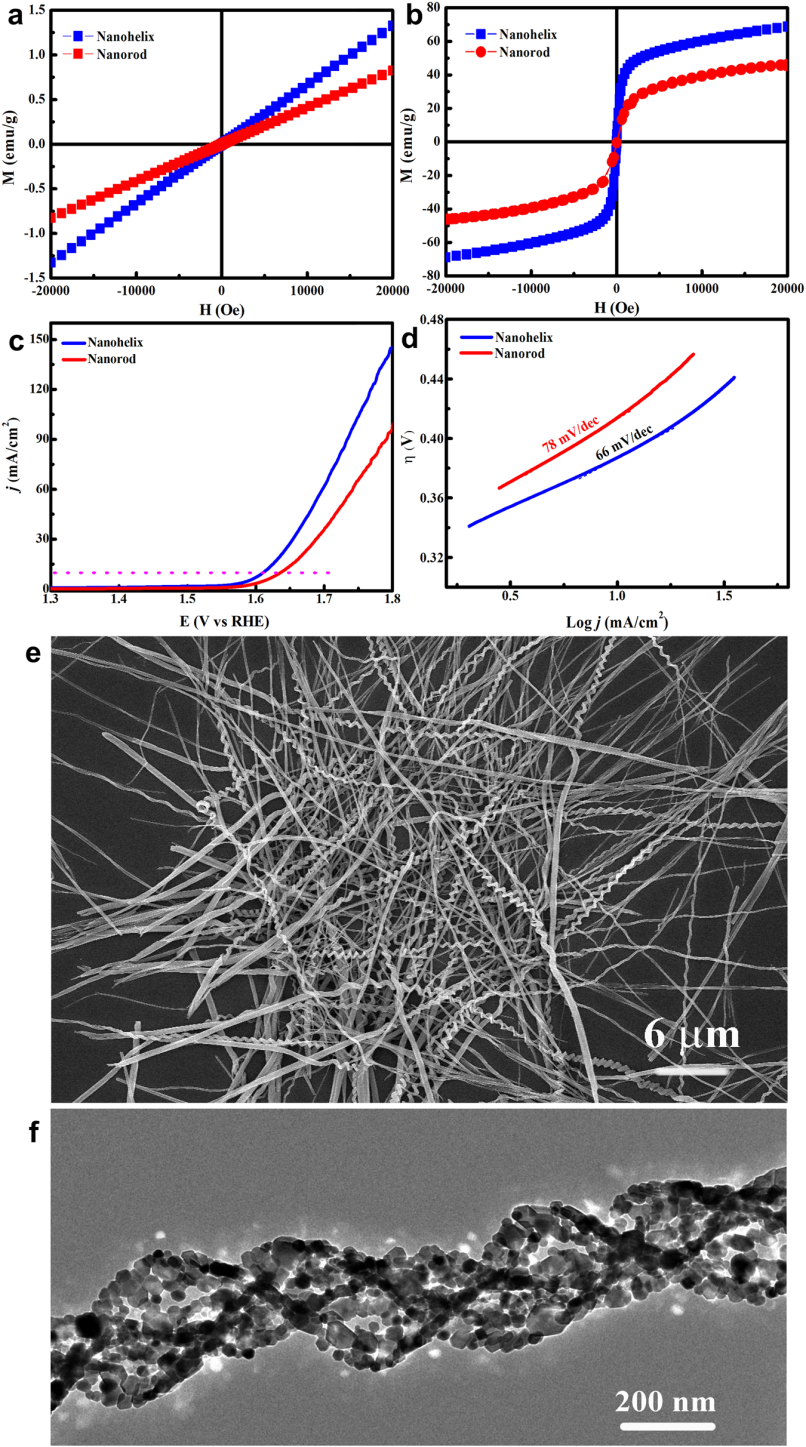
Applications and extensions of the helical complex. **a** M-H curves at 300 K and **b** M-H curves at 2 K of the nanorods and nanohelices; **c** LSV curves and **d** Tafel slopes of the nanorods and nanohelices as electrocatalysts for the oxygen evolution reaction; **e** SEM image of tri-cobalt/nickel salicylate hydroxide hexahydrate nanohelices; **f** TEM image of a polycrystalline Co_3_O_4_ nanohelix.

The structure discrepancy between nanorods and nanohelices brings up the possibility of improving the materials’ properties. Due to their special structure, the nanohelices might have a low anisotropic barrier, and the rotation of the magnetic vector could be relatively easy. Our magnetism study does reveal such differences between the nanorods and nanohelices (Fig. 7a, b). The nanohelices have higher susceptibility than the nanorods, whether at low temperature (2 K) or at room temperature (300 K), which indicates the helical structure a better paramagnetic material. When used as an electrocatalyst for the oxygen evolution reaction (OER), the nanohelices have an onset potential and an overpotential lower than those of the nanorods, as revealed by linear sweep voltammogram (LSV) curves (Fig. 7c). The Tafel slope of the nanohelices is also lower than that of the nanorods (Fig. 7d). These results indicate that the nanohelices possess better OER activity, further revealing the advantage of the helical structure. The performance advantage not only comes from the global effect, such as increased specific surface area, but also comes from the local characteristics of helical structures, such as positive and negative curvature variations. Surely, to deeply understand the structure-property relationship of nanohelices, a lot of further work is needed.

This twisting process has also been applied to the formation of other helical complexes, tri-nickel salicylate hydroxide hexahydrate and tri-cobalt/nickel salicylate hydroxide hexahydrate, by replacing cobalt acetate with the corresponding acetate. SEM image in Fig. 7e shows the one-dimensional helical structure of tri-cobalt/nickel salicylate hydroxide hexahydrate.

Our successful syntheses of Co- and/or Ni-based helices provide an opportunity to prepare inorganic helices through further conversions. The helical structure of the complex can be maintained well during the thermal decomposition process. Several metal oxide nanohelices assembled by Co_3_O_4_, NiO, or NiCo_2_O_4_ nanocrystals have been synthesized. Details about the synthesis process and characterizations are shown in (Supplementary Method 5 and Supplementary Fig. 15). TEM image of a Co_3_O_4_ polycrystalline nanohelix is shown in Fig. 7f. The polycrystalline Co_3_O_4_ helical structure shows better electrochemical performance than its rod-like counterpart and has potential applications in Li-ion batteries (Supplementary Fig. 16).

## Discussion

The multiply reversible twisting transformation between two stable and crystal-type final products of chemical reactions is an ultra-low probability event, which requires very subtle energy balance established between them. Through a large number of experiments, we have found a delicate competition and collaboration in a crystal structure, in which a subtle energy balance between twisting and untwisting products has been established. Such a balance in this new system makes the multiply reversible twisting transformation between nanowires and nanohelices be realized for the first time.

Of course, to achieve this transformation, appropriate reaction conditions are also indispensable. In our experiments, the presence of suitable solvent is very important and could affect crystal structure somehow. The lattice parameters and crystallinity of nanohelices could be changed in a certain range. This effect could be attributed to the special crystal system, in which the subtle energy balance could be slightly affected by suitable solvent, leading to the change in crystal structure.

However, the realization of multiply reversible twisting transformation is not a simple solvent softening process, but a targeted solvothermal reaction to adjust the energy-balanced competition and collaboration in a distinctive system. Both nanohelices and nanowires are stable final products of solvothermal reactions. Which appears depends on the result of such energy-balanced competition and collaboration controlled by the targeted solvothermal conditions.

The whole transformation process with reversible twisting capability is of importance, which not only provides a platform to study the novel behavior of subtly balanced system, but also brings a new perspective in crystallography, enriching the crystallographic theory. It is the key step for the realization of manifold-reversible crystal transformation, which may have wide applications in changeable nanoraster, sensitive nanoswitch, and encryption materials.

In summary, we report the first synthesis of switchable Co-based complex nanohelices through a self-twisting mechanism without the need for any surfactant, template, substrate, or high-temperature process. Theoretical calculations and characterizations demonstrate that the twisting force stems from the competition between the condensation reaction and stacking process, a mechanism quite different from those previously reported for helices. The as-synthesized nanohelices have an unusual characteristic, namely “elasticity,” reflected in the reversible changes of the lattice parameters and the mutual transformation between the nanowires and nanohelices. Compared with their counterpart (nanorods), the produced nanohelices have better properties as a paramagnetic material and electrochemical catalyst. The twisting process can be extended to other complex nanohelices and corresponding polycrystalline inorganic oxide nanohelices through further conversions. The nanohelices with “elasticity” open a door for exquisite and transformable crystals, as well as the consequent investigations of their spectroscopic characteristics and property tailoring. The realization of the twisting–untwisting–retwisting cycle enriches the concept of crystal transformation and provides a new thought for designing reversible processes relying on special mutual effects between molecular interaction and crystal configuration.

## Methods

### Materials

Salicylic acid (C_7_H_6_O_3_) was purchased from Shanghai Lingfeng Chemical Reagent Co., Ltd. Cobaltous acetate tetrahydrate (C_4_H_6_CoO_4_ · 4H_2_O) was purchased from Sinopharm Chemical Reagent Co., Ltd. KOH, ethanol, 1-Hexadecylamine, 3-Hydroxybenzoic acid were purchased from Aladdin Co., Ltd. Catechol, Salicylaldehyde were purchased from Macklin Co., Ltd.

### Synthesis of tri-cobalt salicylate hydroxide hexahydrate nanohelices

In a typical procedure, 0.010 g/mL cobalt acetate tetrahydrate ethanol solution was mixed with an equal volume of 0.015 g/mL salicylic acid ethanol solution under vigorous magnetic stirring to form a purple solution. The purple solution was transferred into a Teflon-lined stainless steel autoclave. The autoclave was then maintained at 80 °C for 4 h. After cooling to room temperature, the precipitate product was collected, washed, and dried at 80 °C in air.

### Supplementary information

Supplementary Information

## Data Availability

The data that support the findings of this study are available within this paper or included in the Supplementary Information. The data are available from the corresponding author upon request.
