# Supplementary material for: Twisting, untwisting, and retwisting of elastic Co-based nanohelices
Source: Nat Commun. 2023 Jul 22;14:4426. doi: 10.1038/s41467-023-40001-w (PMC10363140; doi:10.1038/s41467-023-40001-w)
Supplement: Supplementary file 1 — Supplementary Information [file 41467_2023_40001_MOESM1_ESM.pdf]

Twisting, Untwisting, and Retwisting of Elastic Co-based Nanohelices

Wei Du[#,1,6], Feng Gao[#,*,2], Peng Cui[3], Zhiwu Yu[4], Wei Tong[5], Jihao Wang[3,5], Zhuang Ren[5], Chuang Song[2], Jiaying Xu[1], Haifeng Ma[1], Liyun Dang[1], Di Zhang[1], Qingyou Lu*[,3,5], Jun Jiang*[,3], Junfeng Wang*[,4], Li Pi[3,5], Zhigao Sheng[5], Qingyi Lu*[,1]

[1] State Key Laboratory of Coordination Chemistry, School of Chemistry and Chemical Engineering, Collaborative Innovation Center of Advanced Microstructures, Nanjing National Laboratory of Microstructures, Nanjing University, 210023 Nanjing, P. R. China

[2] Department of Materials Science and Engineering, Jiangsu Key Laboratory of Artificial Functional Materials, Collaborative Innovation Center of Advanced Microstructures, College of Engineering and Applied Science, Nanjing University, 210023 Nanjing, P. R. China

[3] Hefei National Laboratory for Physical Sciences at Microscale and Anhui Laboratory of Advanced Photon Science and Technology, University of Science and Technology of China, 230026 Hefei, AnHui, P. R. China

[4] High Magnetic Field Laboratory, CAS Key Laboratory of High Magnetic Field and Ion Beam Physical Biology, Hefei Institutes of Physical Science, Chinese Academy of Sciences, 230031 Hefei, Anhui, P. R. China

[5] Anhui Province Key Laboratory of Condensed Matter Physics at Extreme Conditions, High Magnetic Field Laboratory and High Magnetic Field Laboratory of Anhui Province, HFIPS, Chinese Academy of Sciences, 230031 Hefei, Anhui, P. R. China

[6] State Key Laboratory of Materials-Oriented Chemical Engineering, College of Chemical Engineering, Nanjing Tech University, 211816 Nanjing, P. R. China

* Corresponding authors

[#] These authors contributed equally to this work.

Supplementary method

1. Synthesis of tri-cobalt salicylate hydroxide hexahydrate nanorods

In a typical procedure, 0.02 g/mL cobalt acetate tetrahydrate solution was mixed with an equal volume of 0.03 g/mL salicylic acid - 0.015 g/mL potassium hydroxide solution under vigorous magnetic stirring at room temperature. Then the precipitate was collected, washed, and dried at 80 °C in air.

2. Synthesis of tri-cobalt salicylate hydroxide hexahydrate nanohelices with the presence of hexadecylamine

In a typical procedure, 0.010 g/mL cobalt acetate tetrahydrate ethanol solution was mixed with an equal volume of 0.015 g/mL salicylic acid ethanol solution under vigorous magnetic stirring to form a purple solution. Then appropriate amount of hexadecylamine was added into the solution under stirring. The mixed solution was transferred into a Teflon-lined stainless steel autoclave. The autoclave was then maintained at 80 °C for 4 h. After cooling to room temperature, the precipitate product was collected, washed, and dried at 80 °C in air.

3. Nanowires straightened from nanohelices with moderate crystallinity

(1) Synthesizing the nanohelices with moderate crystallinity: 0.010 g/mL cobalt acetate tetrahydrate ethanol solution was mixed with an equal volume of 0.015 g/mL salicylic acid ethanol solution under vigorous magnetic stirring to form a purple solution. The purple solution was transferred into a Teflon-lined stainless steel autoclave. The autoclave was then maintained at 80 °C for 1 h. After cooling to room temperature, the precipitate product was collected and washed.

(2) Straightening the nanohelices with moderate crystallinity: The obtained precipitate was dispersed in 30 mL of ethanol, and then the mixture was transferred into a Teflon-lined stainless steel autoclave. The autoclave was maintained at 160 °C for 18 h. After cooling to room temperature, the precipitate was collected, washed, and dried at 80 °C in air.

4. Nanohelices retwisted from nanowires straightened from nanohelices with moderate

crystallinity

(1) Synthesizing the nanohelices with moderate crystallinity: 0.010 g/mL cobalt acetate tetrahydrate ethanol solution was mixed with an equal volume of 0.015 g/mL salicylic acid ethanol solution under vigorous magnetic stirring to form a purple solution. The purple solution was transferred into a Teflon-lined stainless steel autoclave. The autoclave was then maintained at 80 °C for 1 h. After cooling to room temperature, the precipitate product was collected and washed.

(2) Straightening the nanohelices with moderate crystallinity: The obtained precipitate was dispersed in 30 mL of ethanol, and then the mixture was transferred into a Teflon-lined stainless steel autoclave. The autoclave was maintained at 160 °C for 18 h. After cooling to room temperature, the precipitate was collected and washed.

(3) Retwisting the straightened nanowires: The above straightened nanowires were dispersed in 30 mL of ethanol, and the mixture was stirred under ambient conditions for 40 days. Then the mixture was transferred into a Teflon-lined stainless steel autoclave. The autoclave was maintained at 80 °C for 7 days. After cooling to room temperature, the precipitate was collected, washed, and dried at 80 °C in air.

5. Polycrystalline inorganic oxide nanohelices

For Co_3O_4, NiO, or $NiCo_2O_4$ helices, the corresponding precursor — tri-cobalt salicylate hydroxide hexahydrate, tri-nickel salicylate hydroxide hexahydrate, or tri-cobalt/nickel salicylate hydroxide hexahydrate — was heated to 500 °C with a ramping rate of 1 °C/min and then kept at that temperature for 10 min in air. After cooling to room temperature, the black powders were collected for characterizations.

6. Computation methods

The crystal structure of tri-cobalt salicylate hydroxide hexahydrate was optimized at the spin-polarized density functional theory (DFT) level using the Vienna Ab-initio Simulation Package (VASP).[1] The Perdew-Burke-Ernzerhof (PBE) functional within the generalized gradient approximation (GGA) was employed.[2] The GGA+U method was used to describe partially filled

d-orbitals.[3] The long-range van der Waals (vdW) interactions were described by the empirical correction method (DFT-D2).[4] The kinetic energy cutoff was set to be 400 eV, and the Brillouin zone was sampled with 1×1×5 Monkhorst-Pack k-points. XRD simulations were performed by using the Reflex module of the Materials Studio software package of Accelrys Inc based on the optimized geometry. IR spectrum simulations were implemented with Gaussian 09 program[5] based on the geometry of a tri-cobalt hydroxide hexahydrate monomer, using the B3LYP[6] density functional with the 6-31G(d) basis set for C, O, and H, and the effective core potential (ECP) basis set LANL2DZ[7] for Co.

7. EXAFS characterizations

The EXAFS spectra were collected at beamline 14W1 at the Shanghai Synchrotron Radiation Facility (SSRF). The electron storage ring operated at 3.5 GeV under "top-up" mode with a current of 220 mA. A pair of Si(111) crystals were employed for the monochromator; they were detuned slightly to suppress high-order harmonic rejection. Beam energy was calibrated by assigning the first inflection on the K-adsorption edge of Co metal foil to an energy of 7709 eV. A thin ion sample holder was filled with nanorod or nanohelix sample covered with Kapton tape and placed vertically to the incident beam. XAFS data were collected in transmission mode. Data analysis was accomplished using Athena and Artemis. For the X-ray absorption near-edge structure (XANES) analysis, the experimental absorption coefficients as a function of energies μ(E) were processed by background subtraction and normalization procedures and reported as "normalized absorption". For the EXAFS analysis, the raw data were converted from E space to k space and weighted by k^3 to yield a $\chi^3(k)$ function. Then, the $\chi^3(k)$ function was Fourier transformed to R space, where shell-by-shell fitting was done. The amplitude reduction factors, S_0^2, were determined by fitting the experimental data obtained from a Co foil and fixing the Co-Co coordination number (CN) to be 12, followed by fixing for further analysis of the measured samples. The zero-energy point (E_0) and local structure environment, including coordination number (CN), bond distance (R), and Debye-Waller (DW) factor around the absorbing atoms, were allowed to vary during the fitting process.

8. Li-ion battery (LIB) tests of Co_3O_4 nanoparticle-assembled nanohelices and nanorods

The electrochemical testing was carried out in a CR2032-type coin cell. The working electrode was prepared by mixing 80 wt% active material, 10 wt% acetylene black, and 10 wt% polyvinylidene fluoride (PVDF) in N-methylpyrrolidinone (NMP). The formed slurry was coated onto a copper foil. After solvent evaporation at room temperature, the electrode was further dried in a vacuum oven at 80 °C for 12 h. Then the electrode was assembled into coin cells (CR2032) with a Celgard separator membrane and lithium metal as the counter electrode. The electrolyte solution was 1 mol/L $LiPF_6$ in ethylene carbonate (EC)-diethyl carbonate (DEC) (1:1 by volume). The test cells were assembled in an argon-filled (99.999%) glove-box. The galvanostatic charge-discharge and cycling performance measurements were carried out using a LAND CT2001A Battery Testing System (Wuhan Land Electronic Co. Ltd., China) between 0.05 and 3.0 V (vs. Li/Li^+).

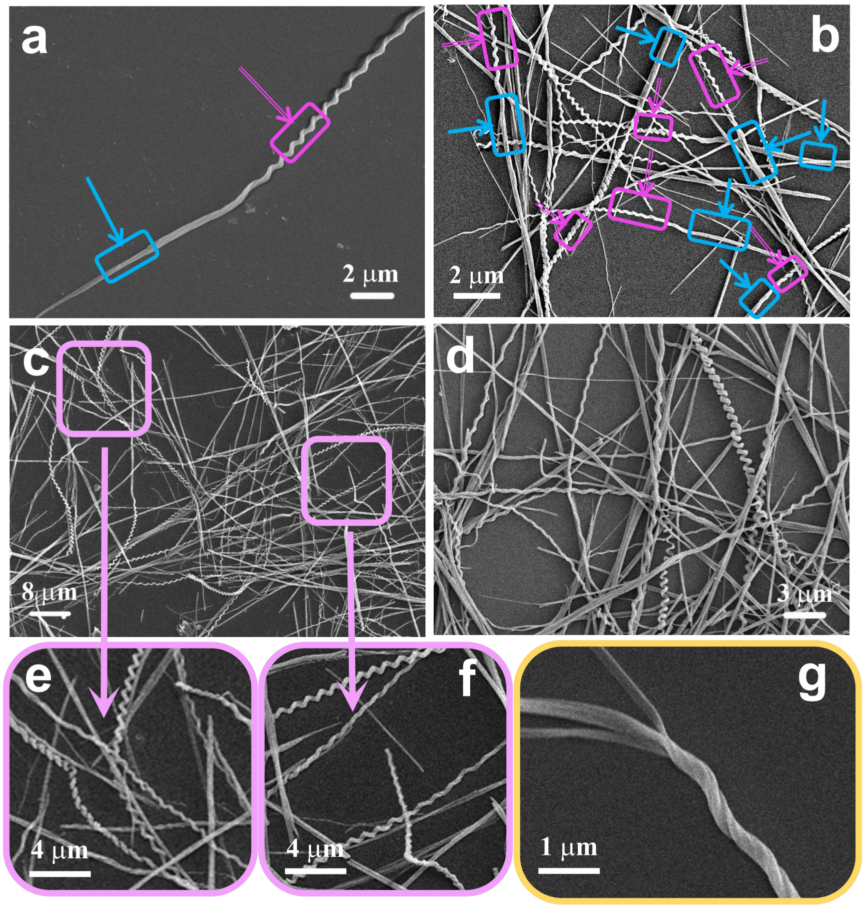

Supplementary Figure 1 (a) SEM image of a single nanohelix: In a single nanohelix, there is a tightly twisted section (purple-red box) and a loosely twisted section (blue box); (b) SEM image of many nanohelices: These nanohelices also have tightly twisted sections (purple-red boxes) and loosely twisted sections (blue boxes); (c~g) SEM images with different magnifications of the as-synthesized nanohelices.

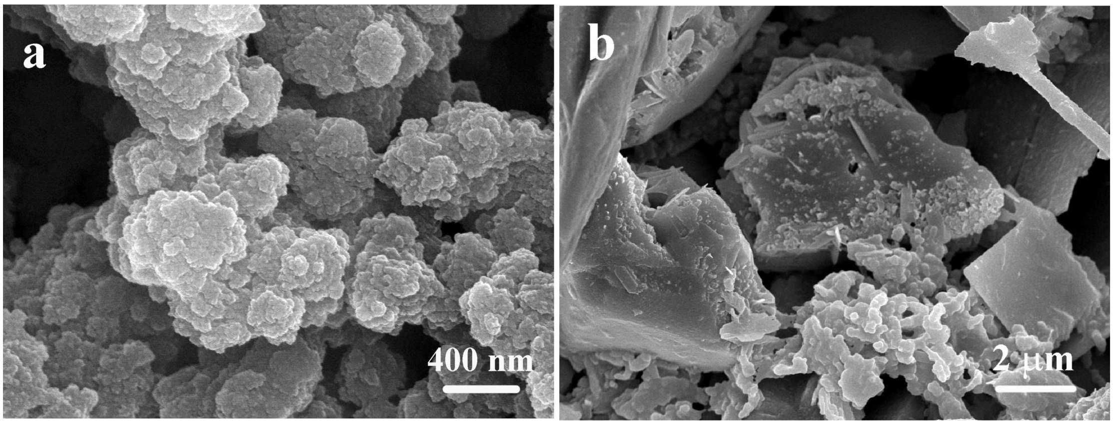

Supplementary Figure 2 SEM images of the samples synthesized with (a) pyrocatechol and (b) m-hydroxybenzoic acid as coordinating agents, respectively, in both of which no nanohelical structures can be found.

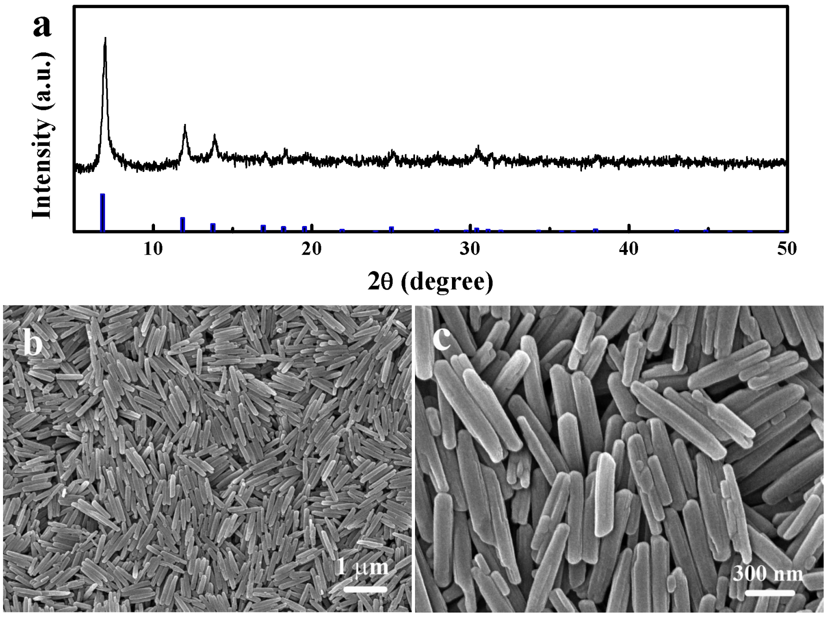

Supplementary Figure 3 (a) XRD pattern and (b, c) SEM images of the tri-cobalt salicylate hydroxide hexahydrate nanorods.

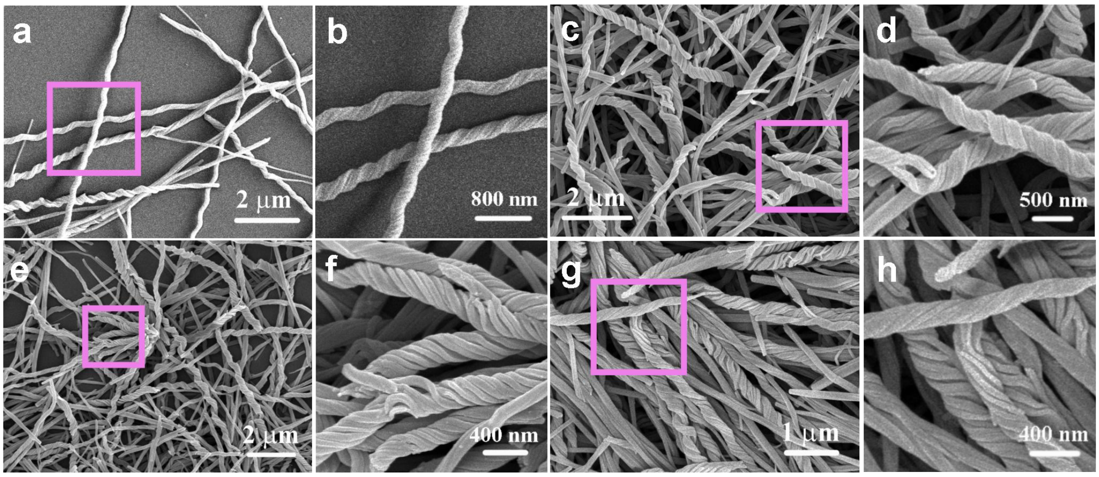

Supplementary Figure 4 (a~h) SEM images with different magnifications of the nanohelices synthesized in the presence of hexadecylamine.

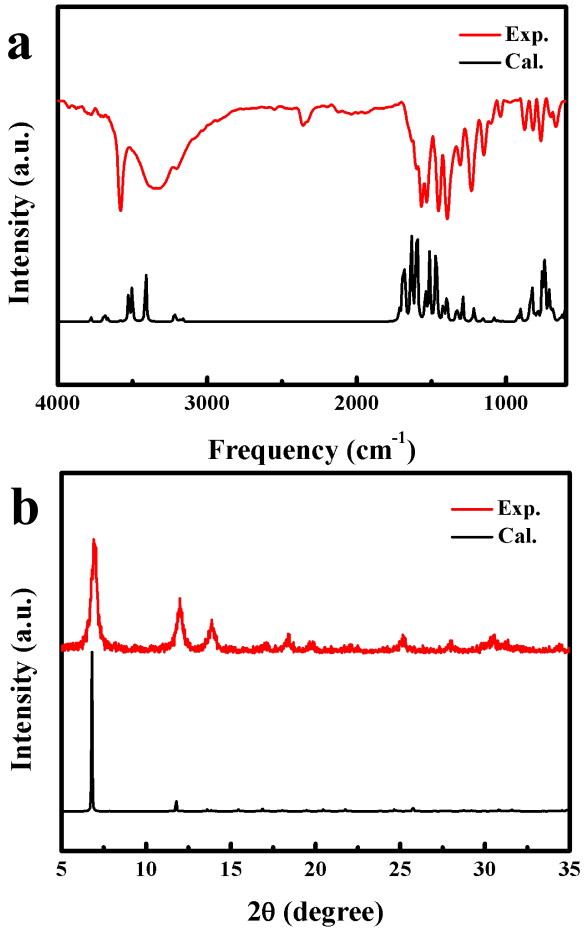

Supplementary Figure 5 (a) IR spectra and (b) XRD patterns of the theoretical model and the experimental sample.

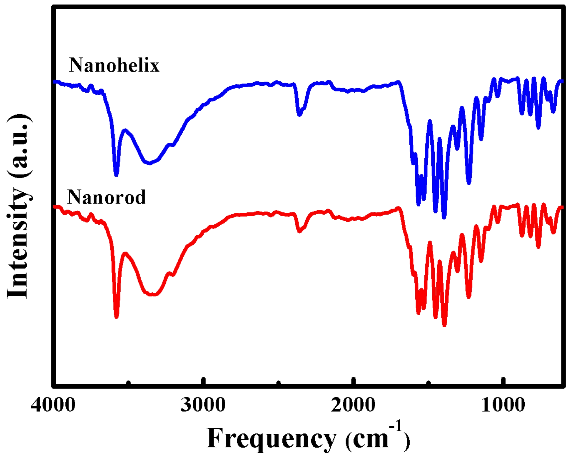

Supplementary Figure 6 IR spectra of the nanorods and nanohelices.

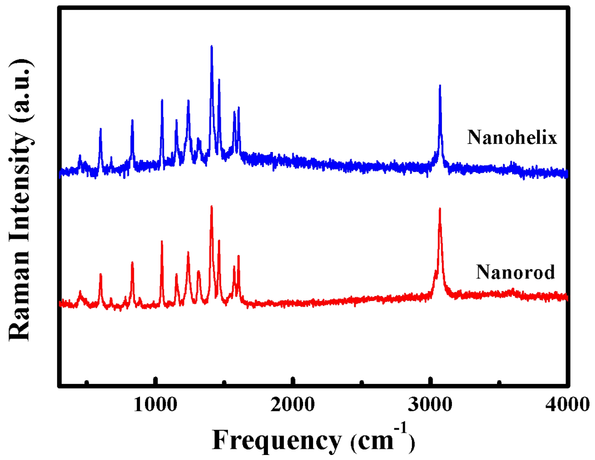

Supplementary Figure 7 Raman spectra of the nanorods and nanohelices.

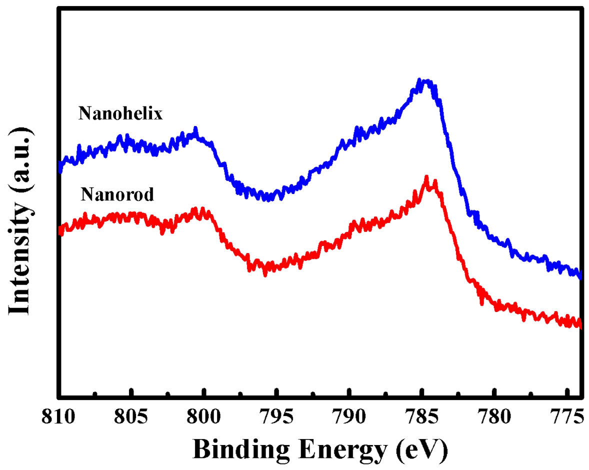

Supplementary Figure 8 XPS spectra of the nanorods and nanohelices.

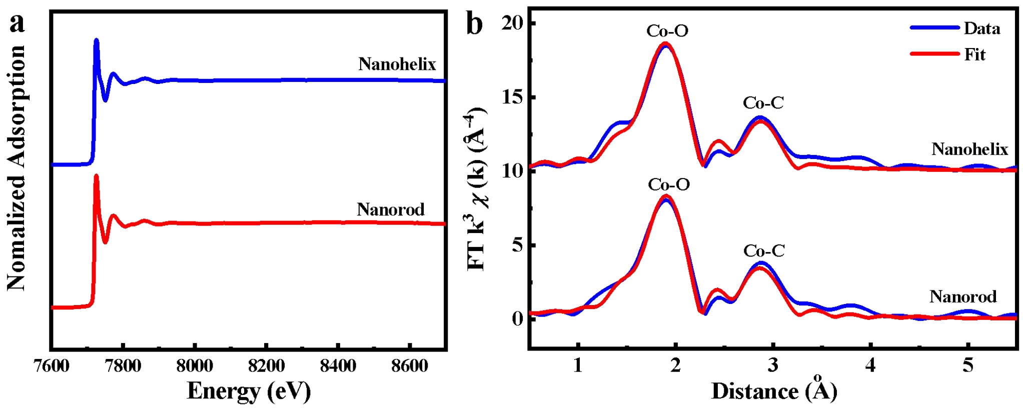

Supplementary Figure 9 (a) XANES profiles and (b) EXAFS R spaces of the nanorods and nanohelices. The XANES profiles in Supplementary Figure 9a exhibit that the cobalt structures in both samples are very similar. Supplementary 9b displays the EXAFS data of both samples, as well as the fitting results. The corresponding fitting results in Supplementary Table 1 clearly demonstrate the first shell of Co−O distance (R) is centered at 2.05 Å with a Co−O coordination number (CN) of 6, which is in good agreement with the theoretical model (CN = 6). The second shell of Co−Co can also be determined by EXAFS, and Co−Co distance (R) is centered at 3.04 Å with a Co−Co coordination number (CN) of 3. The EXAFS spectra of the nanorods and nanohelices are more or less identical, indicating that they have the similar local chemical structures.

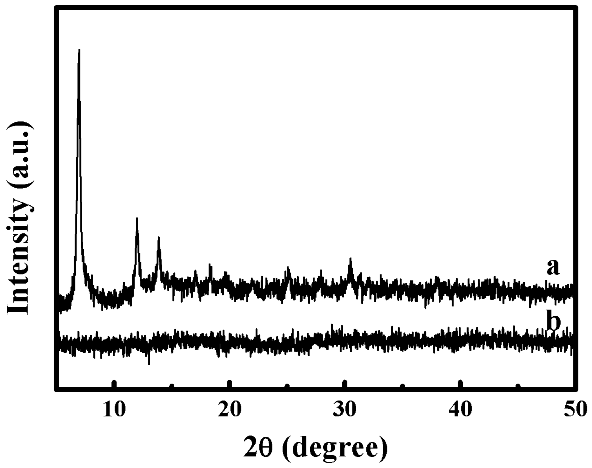

Supplementary Figure 10 XRD patterns of the nanorods (a) before and (b) after soaking in ethanol for 24 h.

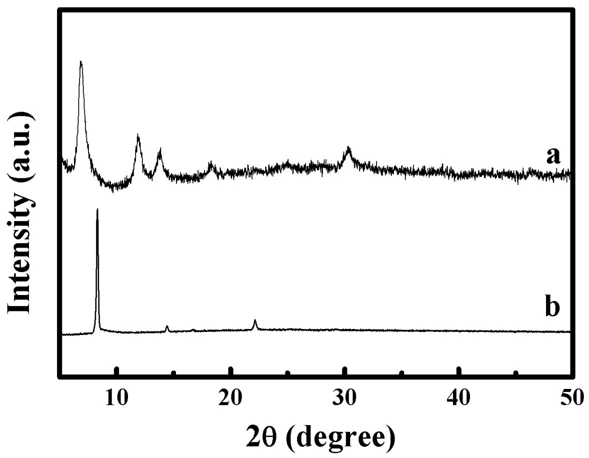

Supplementary Figure 11 XRD patterns of the nanohelices synthesized through the typical synthesis procedure (a) with 80 °C drying step and (b) dried naturally in air.

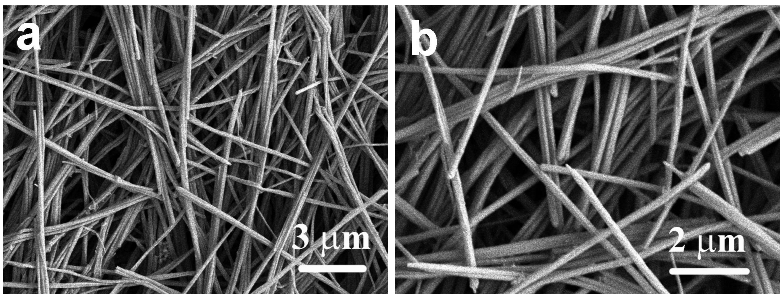

Supplementary Figure 12 (a, b) SEM images of nanowires straightened from nanohelices with moderate crystallinity.

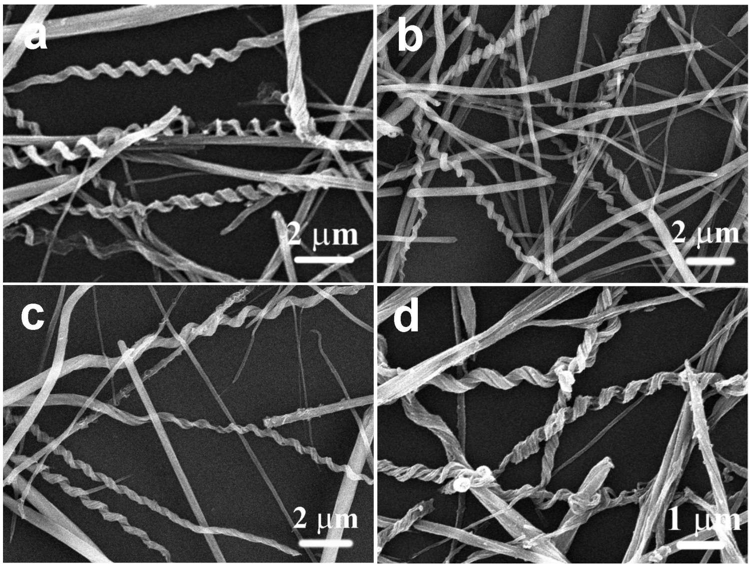

Supplementary Figure 13 (a~d) SEM images of nanohelices retwisted from nanowires straightened from nanohelices with moderate crystallinity.

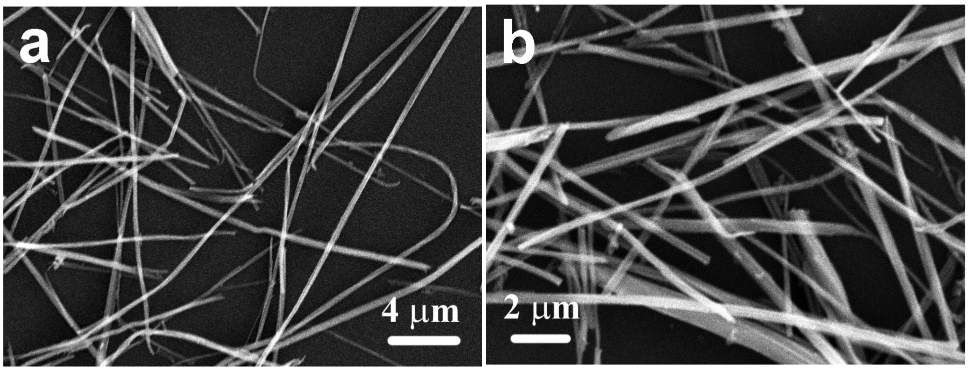

Supplementary Figure 14 (a, b) SEM images of nanowires straightened from nanohelices with high crystallinity.

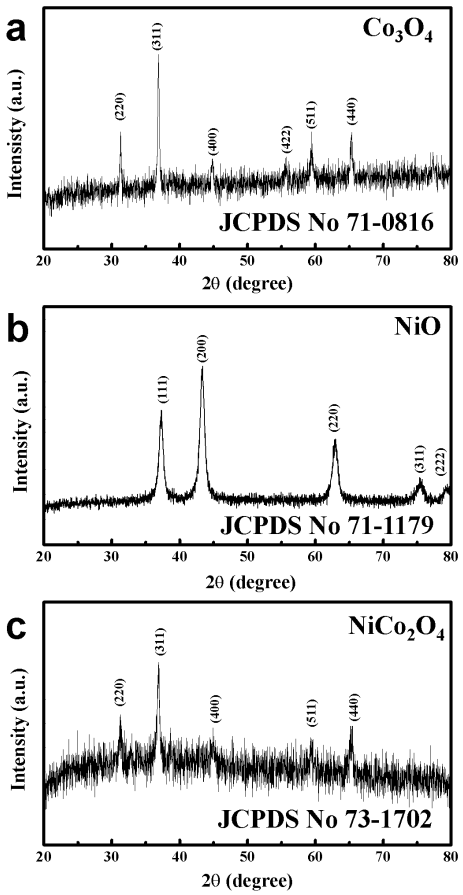

Supplementary Figure 15 XRD patterns of the polycrystalline inorganic oxide nanohelices obtained by calcining (a) Tri-cobalt salicylate hydroxide hexahydrate; (b) Tri-nickel salicylate hydroxide hexahydrate; (c) Tri-cobalt/nickel salicylate hydroxide hexahydrate.

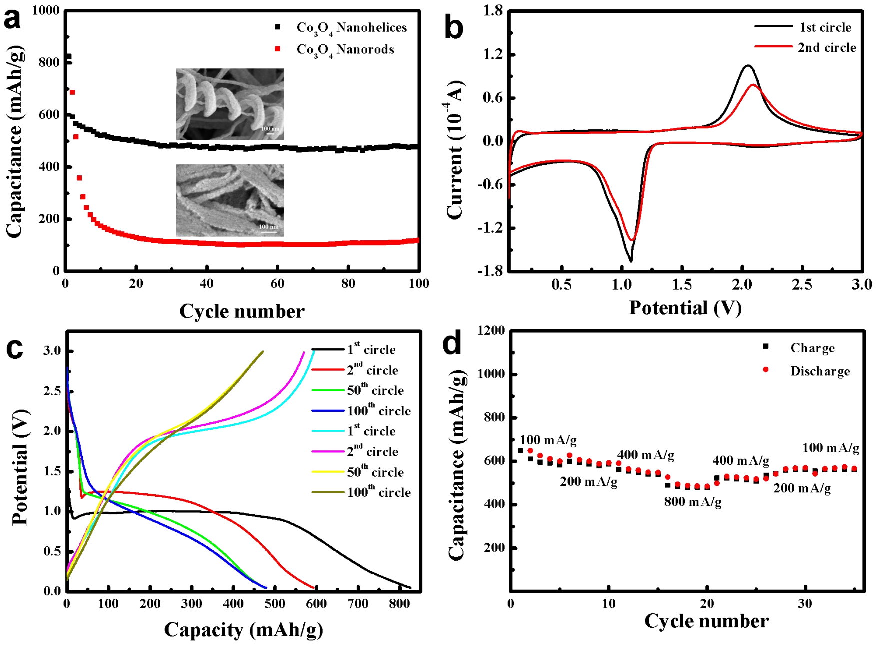

Supplementary Figure 16 (a) Cyclic test of Co_3O_4 nanohelix-based LIB compared to Co_3O_4 nanorod-based LIB; (b) Cyclic voltammetry curves, (c) Charge-discharge curves, and (d) Cyclic performance at different current densities of Co_3O_4 nanohelix-based LIB.

Supplementary Table 1 Co K-edge EXAFS fitting results of the nanorods and nanohelices (*R*: distance; *CN*: coordination number; $ϭ^2$: Debye-Waller factor; ΔE_0: inner potential correction)

Sample	Co-O (first shell)		Co-Co (second shell)		$ϭ^2$ (Å^2)	ΔE_0 (eV)
	R (Å)	CN	R (Å)	CN		
Nanorods	2.050 ± 0.007	5.9 ± 0.5	3.041 ± 0.016	3.4 ± 1.0	0.006 ±0 .001	-0.2 ± 0.7
Nanohelices	2.049 ± 0.008	6.3 ± 0.5	3.040 ± 0.022	3.1 ± 1.1	0.012 ± 0.001	-0.3 ± 0.9

Supplementary References

1. Kresse, G. & Furthmuller. J. Efficiency of ab-initio total energy calculations for metals and semiconductors using a plane-wave basis set. *J. Comput. Mater. Sci.* **6,** 15-50 (1996).
2. Sartorel, A. *et al*. Water oxidation at a tetraruthenate core stabilized by polyoxometalate ligands: experimental and computational evidence to trace the competent intermediates. *J. Am. Chem. Soc.* **131,** 16051-16053 (2009).
3. Zhou, J. & Sun, Q. Magnetism of phthalocyanine-based organometallic single porous sheet. *J. Am. Chem. Soc.* **133,** 15113-15119 (2011).
4. Grimme, S. Semiempirical GGA-type density functional constructed with a long-range dispersion correction. *J. Comput. Chem.* **27,** 1787-1799 (2006).
5. Frisch, M. J. *et al*. *Gaussian 09, Revision D.01*, Gaussian, Inc.: Wallingford, CT, USA, 2009.
6. Zhang, I. Y., Wu, J. & Xu, X. Extending the reliability and applicability of B3LYP. *Chem. Commun.* **46,** 3057-3070 (2010).
7. Xu, X. & Truhlar, D. G. Performance of effective core potentials for density functional calculations on 3d transition metals. *J. Chem. Theory Comput.* **8,** 80-90 (2012).
